# A Comparative Analysis of Economic Cost of Podoconiosis and Leprosy on Affected Households in the Northwest Region of Cameroon

**DOI:** 10.4269/ajtmh.17-0931

**Published:** 2018-02-19

**Authors:** Ayok M. Tembei, Jonas A. Kengne-Ouaffo, Elvis A. Ngoh, Bonekeh John, Theobald M. Nji, Kebede Deribe, Peter Enyong, Theresa Nkuo-Akenji, Gail Davey, Samuel Wanji

**Affiliations:** 1 Epidemiology and Control of Infectious Diseases, Department of Microbiology and Parasitology, University of Buea, Buea, Cameroon;; 2 Research Foundation in Tropical Diseases and Environment, Buea, Cameroon;; 3 Mbebah Vigilantic Farming and Development Association in Ndop, Ngokutunja Sub-division of the North West Region, Ndop, Cameroon;; 4 Department of Sociology and Anthropology, University of Buea, Buea, Cameroon;; 5 Wellcome Trust Centre for Global Health Research, Brighton and Sussex Medical School, Brighton, United Kingdom;; 6 School of Public Health, Addis Ababa University, Addis Ababa, Ethiopia

## Abstract

Leprosy and podoconiosis (podo) are neglected tropical diseases that cause severe disfigurement and disability, and may lead to catastrophic health expenditure and hinder economic development of affected persons and households. This study compared economic costs of both diseases on affected households with unaffected neighboring households in the Northwest Region (N.W.R.) of Cameroon. A matched comparative cross-sectional design was used enrolling 170 households (43 podo case households, 41 podo control households, 43 leprosy case households, and 43 leprosy control households) from three health districts in the N.W.R. Direct treatment costs for podo averaged 142 United State dollar (USD), compared with zero for leprosy (*P* < 0.001). This was also reflected in the proportion of annual household income consumed (0.4 versus 0.0, respectively, *P* < 0.001). Both diseases caused considerable reductions in working days (leprosy 115 versus podo 135 days. *P* for comparison < 0.001). The average household income was considerably lower in podo-affected households than unaffected households (410 versus 913 USD, *P* = 0.01), whereas income of leprosy-affected households was comparable to unaffected households (329 versus 399 USD, *P* = 0.23). Both leprosy and podo cause financial burdens on affected households, but those on podo-affected families are much greater. These burdens occur through direct treatment costs and reduced ability to work. Improved access to public health interventions for podo including prevention, morbidity management and disability prevention are likely to result in economic returns to affected families. In Cameroon, one approach to this would be through subsidized health insurance for these economically vulnerable households.

## INTRODUCTION

Neglected tropical diseases (NTDs) are chronic, disabling, and disfiguring conditions commonly occurring in settings of extreme poverty, particularly in the rural poor and some disadvantaged urban populations.^1^ Neglected tropical diseases are both the consequence and cause of poverty. They are common among very poor individuals and they cause poverty, through stigma, disability, and reduced productivity. The world’s greatest concentration of poverty occurs in sub-Saharan Africa (SSA). The World Bank analyzed 51% of the population of SSA as living on less than 1.25 United State dollar (USD) per day, and 73% of the population living on less than 2 USD per day.^2^ Leprosy and podoconiosis (podo) are diseases that hinder economic development and cause chronic life-long disability in the poor and disenfranchised communities in which they are most prevalent.

Podoconiosis (endemic nonfilarial elephantiasis) is a noninfectious geochemical disease caused by the conjunction of environmental, genetic, and economic factors.^3^ This condition, which has been categorized as an environmental geochemical disease resulting from irritant soil, occurs in individuals who have been exposed to red clay soil derived from alkaline volcanic rock.^4^ Podoconiosis has been reported among barefoot farmers in volcanic highland zones of Africa, Central and South America, and northwestern India.^4^ In Cameroon, podo was first described in 1981 by Price.^5^ An estimated 500,000 persons are affected with podo in the highland zones of Cameroon.^4^ Despite the public health importance of the disease, it has received little attention from policy makers in Cameroon. This may partly be because of the absence of data on the economic impact of the disease in this country.

The disease is characterized by bilateral swelling of the lower legs with mossy and nodular changes to the skin.^6^ Podoconiosis follows a chronic course, with progressively increasing disability, especially with continued exposure to irritant soils. However, with simple treatment, the condition is reversible. The disability and deformity caused by podo have been shown to have economic consequences^7^ just like leprosy.^8^

Leprosy, also known as Hansen’s disease, is a chronic infectious disease caused by a slow-growing bacterium called *Mycobacterium leprae*. From the World Health Organization’s end of first quarter 2017 reports, 171,948 cases were registered as receiving multidrug therapy, with a registered prevalence rate of 0.23 per 10,000 population^7^ from 143 countries.^9^ The Americas and southeast regions registered the highest number of leprosy cases. A total of 214,783 new cases were reported from 143 countries during 2016, corresponding to the global new-case detection rate of 2.9 per 100,000 population.^9^

Although leprosy has been eliminated as a public health problem in Cameroon, there are still areas with high prevalence such as Essimbiland (Menchum Division) with a prevalence of 4.5/10,000 and Mbingo (Boyo Division) with a prevalence of 3.5/10,000 in the Northwest Region (N.W.R.).^10^

Leprosy affects mainly the skin and peripheral nerves. Its diagnosis is established based on skin and neurologic examination of the patient.^11^ Without early diagnosis and treatment, leprosy progressively results in physical disabilities.^11^ It is highly contagious, but its morbidity is low because a large portion of the population is naturally resistant to this disease. Transmission has been associated with close and repeated contact with nose and mouth droplets from untreated leprosy patients, and children are more likely to contract the disease than adults.

Both podo and leprosy are diseases associated with devastating disabilities which are likely to interfere with economic and domestic (household chores and leisure) activities, thereby reducing such patients’ ability to attain good health.^12^ According to a study conducted in Ethiopia,^7^ the economic costs of podo in Ethiopia are high, with direct treatment costs being equivalent to 143 USD per patient per year. In addition, the physical challenges of the condition contribute to large productivity losses as most of the patients are of working age.^13^ Given the lack of evidence around the economic impact of podo, the Cameroon government has prioritized diseases with clearer evidence of economic and development impact such as leprosy. There is therefore a need for evidence-based research focusing on the treatment costs and cost burden of podo to fill the gap in the scientific literature. Quantifying the economic impact of podo and comparing this with the impact of leprosy will help underscore the public health importance of podo.

## MATERIALS AND METHODS

### Study design.

The study used a matched comparative cross-sectional study design to estimate the yearly household income and expenditures associated with treatment of diseases (podo, leprosy, and other common conditions) for both affected and unaffected households. A comparative design was used to compare the economic cost of disease between podo- and leprosy-affected households. A semistructured questionnaire was used for data collection documenting economic characteristics of households.

### Study area.

The N.W.R. of Cameroon has 19 health districts with the Batibo and Ndop health districts being the most affected with podo (Wanji et al., unpublished data). The population of Batibo and Ndop practice high levels of subsistence agriculture, which is the main economic driver within both communities. The Ndop plain is known in the region for the cultivation of rice in marshy wetlands. These agricultural factors predispose the inhabitants to the risk of acquiring podo due to continuous exposure of bare feet to the soil. The Mbingo Baptist leprosarium is one of the oldest leprosaria in Cameroon and is in the Fundong health district within the Mejang health area. A good number of leprosy resident households are found in this area owing to referrals from other regions of the country to this leprosarium. Data collection spanned from July to August 2015 within these areas.

### Sampling and study subjects.

Batibo and Ndop health districts were purposively chosen for known high prevalence of podo in previous studies conducted within the N.W.R.^4^ Households with confirmed podo cases living in communities within Batibo and Ndop health districts were sequentially selected from a list of confirmed cases from an earlier prevalence study. Leprosy patients (currently on treatment or treated in the past) living within communities in Mejang health area were sequentially selected from the Mbingo leprosarium register.

Participants consisted of podo and leprosy cases in the most affected and economically active age group (15 years and older) regardless of coexistence of any other disease because economic cost of other diseases was assessed in this study to control for confounders. Controls were individuals from households unaffected by podo or leprosy within the communities of interest. These individuals were matched to cases by age (±5 years), gender, and occupation, and enrolled in a 1:1 case:control ratio.

### Variables and cost estimation.

The prevalence-based model, which quantifies economic costs due to illness occurring within a given time period^13^ (1 year in this study), was used from the societal perspective. Variables for measurement included household annual income, annual economic cost of treatment, and cost burden of diseases for both case and control households. Economic costs were estimated in terms of direct and indirect health costs. Indicators for direct (out-of-pocket) costs and indirect (productivity loss) patient costs were defined, and for analytic purposes, direct costs were divided into categories (Table 1). In this study, direct costs were defined as the medical and nonmedical costs of receiving health care for both patients and accompanying persons. Indirect costs were measured in terms of productive time lost (converted into cost based on the hourly gross income of each household yearly).

**Table 1:** Categories for cost assessment

Cost type	Cost category	Definition
Direct costs (out-of-pocket payments)	Biomedical expenses and hygiene costs	These costs were related to patients’ personal hygiene when taking care of the wounds (e.g., bleach and soap to wash bandages and clothing), irregular expenses for extra medication (e.g., for pain relief), and official fees during treatment.
	Food, lodging, and transportation costs	Food costs included extra meals taken at local food stands by patients or caretakers. Transportation costs refer to the costs of transport for the patient, caretaker(s), and other household members when traveling to and from the hospital to visit the hospitalized patient or when seeking care. Lodging costs included extra rent in the location of the hospital for caretakers.
	Miscellaneous costs	These included a variety of non-systematic costs such as extra phone calls, debts to community workgroups due to illness, gifts to hospitalized patients, extra food from home to hospital, etc.
Indirect costs (disability)	Productivity loss	Productivity loss was based on the calculation of the individual’s (patient and/or caretaker) earnings per calendar year and the percentage of these earnings that was lost because of the morbidity and disability time caused by illness episode or caretaking.

The aforementioned economic variables were estimated for commonly occurring diseases and for leprosy or podo for case households, but for commonly occurring diseases only for control households.

**Figure 1.**
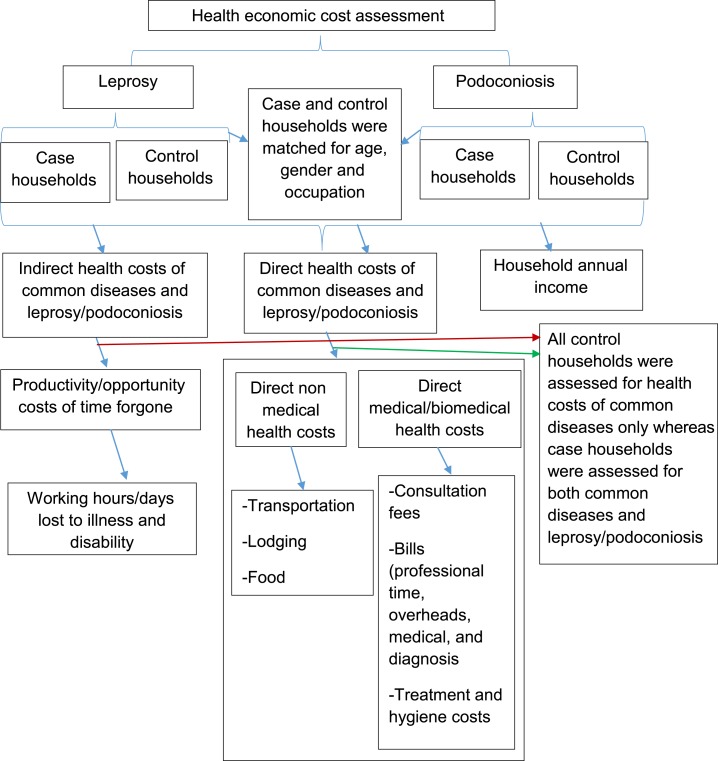
Case–control study design showing health cost assessment method for podoconiosis and leprosy. Common diseases referred to diseases of which every household within the community was likely to observe an episode yearly. This figure appears in color at www.ajtmh.org.

Household earnings were estimated based on the combined salaries of household members participating in the study over the last month and extrapolated to a calendar year, plus the total annual agricultural products multiplied by average market price, to give an annual per household sum (Figure 1).

### Instrument for measurement.

Assessment methods for both case and control households were the same to allow comparison between groups. For both case and control households, a semistructured questionnaire was used to establish sociodemographic and economic characteristics of the household such as direct health cost and economic productivity loss. The questionnaire documented the following:

- Sociodemographic characteristics (age, gender, level of education, number of household members, and marital status) of the case or control individual;
- Yearly income of households (estimated as described previously, and using a list of agricultural products typical of the community);
- Direct payments for nonmedical items such as transportation, lodging, and food for patients and accompanying persons;
- Direct costs incurred for biomedical and medical items which includes professional time, treatment, and overheads in and outside the health facility,^14^ including traditional healers;
- Cost of lost productivity referring to the value of foregone earnings from economic activities as the result of working fewer hours during illness than healthy matched individuals.

An open-ended question was used to estimate other aspects of costing such as gifts resulting from illness, extra phone calls, and extra food from home to treatment site for patient and caretakers.

Patients and their caretakers were asked to estimate their daily hours of work when well and when unwell, to estimate the number of productive hours lost by patients and caretakers because of illness. This was then converted to cost by multiplying the household hourly income (calculated from the annual household income) and the total number of productive hours lost. Efforts were made to reduce the possibility of recall bias by prompting questions from monthly average estimates, then to yearly estimates.

### Households as unit of measurement.

We took the household as the unit of measurement for several reasons. First, the household is the basic economic unit when coping with the illness costs of its members. Second, decisions about treatment are based on negotiations within the household (although not necessarily from an equal bargaining position). Third, the costs of illness reach beyond the sick to involve other household members who care for them and accompany them to seek treatment.

### Data analysis.

The variables from this quantitative study were precoded and data computerized using Epi Info v.3.5 (CDC, Atlanta, GA) and imported to SPSS v.20 (IBM Corp., Armonk, NY) for analysis. The χ^2^ test was used to establish sociodemographic differences between case and control groups. The Kolmogorov–Smirnov test was used to determine mean differences in number of working days lost between case and control households and the normality of the distribution. The Mann–Whitney *U* test of significance was used to determine differences in income status and proportions of health burden between study groups for data not normally distributed owing to outliers.

### Ethical consideration.

Ethical approval was obtained from the University of Buea, Faculty of Health Science Institutional Review Board and the Bamenda Regional Hospital Institutional Review Board after a departmental authorization was issued from the University of Buea. Regional and administrative clearances were obtained from the North West Regional Delegation of Public Health and from district medical officers of health districts. Permission was equally obtained from the Cameroon Baptist Convention (CBC) Health Service to collect data at the Mbingo Baptist hospital. Written informed consent forms were used to ensure participants’ willingness to participate in the study using methods described by Kengne-Ouafo et al.^15^ from their study on perceptions of consent, permission structures, and approaches to the community in Northwest Cameroon.

## RESULTS

### Basic characteristics of participants.

The study enrolled a total of 170 households, 43 affected by podo (and 43 control households matched to the podo proband) and 43 affected by leprosy (and 41 control households matched to the leprosy proband). The discrepancy in cases and controls for leprosy was because of the fact that matched controls for two leprosy patients with respect to gender, age, and occupation could not be found.

The study had an approximate distribution of gender as follows: 48.8% of leprosy cases and controls were female, whereas 48.8% of podo cases and 46.3% of podo controls were female (Table 2). The age of podo participants ranged from 30 to 87 for cases and from 26 to 85 for controls with a mean (standard deviation [SD]) age of 59 (16.8) and 58 (17.3) years for cases and controls, respectively. The age range of leprosy participants was from 27 to 87 for cases and from 25 to 90 for controls with a mean (SD) age of 61 (16) and 61 (15.6) years cases and controls, respectively. Of the 170 respondents, 5.3% (9) attained higher education, 9.4% (16) had secondary education, 58.8% (100) had First School Leaving Certificates, 24.7% (42) did not attain any education, and 1.8% (3) attended other informal educational setups such as adult school and some vocational training.

**Table 2:** Basic characteristics of participants

Variable	Category	Podoconiosis participants (*n* = 84)			Leprosy participants (*n* = 86)		
		Cases (%)	Controls (%)	*P* value	Cases (%)	Controls (%)	*P* value
Gender	Male	22 (51.2)	22 (53.7)	NS	22 (51.2)	22 (51.2)	NS
	Female	21 (48.8)	19 (46.3)		21 (48.8)	21 (48.8)	
Age (years)	Mean (SD)	59 (16.8)	58 (17.3)	NA	61 (16)	61 (15.6)	NA
	Range (Min.–Max.)	57 (30–87)	59 (26–85)		60 (27–87)	65 (25–90)	
Literacy level	Higher	3 (7.0)	4 (10.0)	NS	1 (2.3)	1 (2.4)	NS
	Secondary	4 (9.3)	3 (7.5)		3 (7.0)	6 (14.6)	
	Primary	23 (53.5)	29 (72.5)		25 (58.1)	23 (56.1)	
	None	13 (30.2)	4 (10.0)		14 (32.6)	11 (26.8)	
Marital status	Single	3 (7.0)	3 (7.3)	NS	10 (23.3)	7 (16.7)	NS
	Married/in union	23 (53.5)	30 (73.2)		15 (34.9)	26 (61.9)	
	Divorced/separated	3 (7.0)	1 (2.4)		6 (14.0)	0 (0.0)	
	Widowed	14 (32.6)	7 (17.1)		12 (27.9)	9 (21.4)	
Religion	Christian	43 (100)	41 (100)	NA	43 (100)	42 (100)	NA

Max. = maximium; Min. = minimium; *n* = total sample size; NA = not applicable; NS = not significant; SD = standard deviation; % = percentage. Level of significance; *P* value <0.05.

The marital status among participants showed 13.6% (23) to be single, 55.6% (94) married, 24.9% (42) widows, and 5.9% (10) divorced, with fewer podo patients and leprosy patients being married than their controls. Christianity was the leading religion among participant households for both diseases and their controls.

### Household income, direct and indirect cost of common household diseases.

The average household income for leprosy case households was 329 USD and 399 USD for control households (*P* > 0.05) (current exchange rate (September 10, 2015; 1 USD = 586 FCFA). On the other hand, the yearly average income for podo case household was 410 USD and 913 USD for control households (*P* < 0.05).

Table 3 shows that podo case households spent 33% of their annual income on treatment of commonly occurring diseases, whereas the equivalent figure for control households was 13% (*P* < 0.05). Leprosy case households spent 6% of their annual income and control households 12% on treatment of these common diseases. A mean of 36 and 23 working days were lost each year for leprosy case and control households, respectively, with a statistical value of *P* < 0.05 (95% confidence interval [CI], Kolmogorov–Smirnov test). The working days lost accounted for an average household income loss of 6 USD for case households and 11 USD for control households (*P* > 0.05, 95% CI, Mann–Whitney *U* test). A mean of 54 and 47 working days were lost annually for podo case and control households, respectively, because of commonly occurring diseases (*P* > 0.05, 95% CI). This loss amounted to a yearly average household income loss of 12 USD for case households and 18 USD for control households (*P* > 0.05, 95% CI).

**Table 3:** Household income, direct and indirect cost of common household diseases

Variable	Leprosy respondents (*n*) = 86				Podoconiosis respondents (*n*) = 84		
		*n*	Median ± SD†	*P* value	*n*	Median ± SD	*P* value
Household income	Controls	43	399 (1,146)	NS	41	913 (1,120)	0.005*
	Cases	43	329 (556)		43	410 (1,194)	
Total direct cost common diseases	Controls	43	76 ± 91	NS	40	142 ± 189	NS
	Cases	43	36 ± 75		42	147 ± 183	
	Total	86	56 ± 83	–	82	145 ± 186	–
Cost burden (fraction of household income consumed) (%)	Controls	43	12 ± 78	NS	41	13 ± 55	0.006*
	Cases	43	6 ± 81		42	33 ± 348	
			Mean			Mean	
Working days lost to common diseases	Controls	43	23 ± 19	**0.021**	41	47 ± 53	NS
	Cases	43	36 ± 37		41	58 ± 52	
			Median			Median	
Total indirect cost of common diseases	Controls	43	11 ± 26	NS	41	18 ± 38	NS
	Cases	43	6 ± 14		41	12 ± 24	
	Total	86	9 ± 20	–	82	15 ± 31	–

*n* = sample size; NS = not significant; SD = standard deviation; % = percentage.

* Significant.

† All cost was measured in United State dollar.

### Direct and indirect cost of podo and leprosy on affected households.

Podoconiosis patients spent an average of 137 USD out of pocket on medical treatment of the disease, whereas leprosy patients spent almost nothing for treatment (*P* < 0.05, 95% CI, Mann–Whitney *U* test). Within the nonmedical cost category, podo patients spent a total average cost of 9 USD for transportation, extra food, and rent, whereas leprosy patients spent approximately 0 USD (*P* < 0.05, 95% CI). For miscellaneous costs, podo patients spent an average sum of 9 USD for extra phone calls because of disease and extra food for patients and caretakers. Leprosy patients on the other hand, spent almost nothing for calls and food, (*P* < 0.05).

Overall, podo patients spent an average of 142 USD annually for direct out-of-pocket treatment of podo, whereas leprosy patients spend almost nothing, *P* < 0.05. Podoconiosis patients incurred an annual debt of 34 USD for treatment of podo, thus, 34 USD of the 142 USD spent per annum is money borrowed from friends, family, or community groups which was not the case for leprosy households. At 34.7% of annual income, average out-of-pocket expenditure for podo households clearly exceeded the level defined as catastrophic health expenditure (> 10% of income).

A total of 115 working days were lost for leprosy and 135 days for podo over the past year (*P* < 0.05, Kolmogorov–Smirnov test) (Table 4). These lost days accounted for an annual total loss of 26 USD and 40 USD for leprosy and podo households, respectively (*P* < 0.05, 95% CI, Mann–Whitney *U* test).

**Table 4:** Direct and indirect cost of podo and leprosy on affected households‡

Cost categories	Leprosy (median ± SD)†	Podo (median ± SD)†	*P* value
Direct medical costs	0.00 ± 8	137 ± 488	< 0.001*
Direct nonmedical costs	0.00 ± 0.13	9 ± 24	< 0.001*
Miscellaneous	0.00 ± 0.34	9 ± 18	< 0.001*
Total direct costs	0.00 ± 8	142 ± 517	< 0.001*
Amount borrowed	0.00 ± 8	34 ± 489	< 0.001*
	Mean ± SD	Mean ± SD	
Working days lost	115 ± 62	135 ± 106	< 0.001*
	Median ± SD	Median ± SD	
Total indirect costs	26 ± 45	40 ± 124	NS
Annual economic cost (direct and indirect) of podo/leprosy	26 ± 45	203 ± 559	< 0.001*
Fraction of household income consumed yearly (cost burden) (%)	0 ± 4	40 ± 303	< 0.001*

NS = not significant; Podo = podoconiosis; SD = standard deviation; % = percentage.

* Significant.

† All costs were measured in United States dollar.

‡ Sample size was 86 (43 podo and 43 leprosy).

### Economic cost and cost burden of podo and leprosy.

The annual economic cost of leprosy amounted to a total of 26 USD, whereas that of podo was 203 USD (*P* < 0.05, 95% CI, Mann–Whitney *U* test) (Table 4). Forty percentage of annual household income was used for podo treatment in podo households, whereas almost no household income was used for leprosy treatment (*P* < 0.05, 95% CI).

## DISCUSSION

This study quantifies the economic burden of leprosy and podo in the N.W.R. of Cameroon. Podoconiosis leads to significant financial consequences on affected households, through direct and indirect treatment costs, whereas leprosy has a smaller, but still important, financial impact. In addition, both diseases cause significant loss of productive days per annum. Given the World Bank definition of catastrophic health expenditure as out-of-pocket expenditure of > 10% monthly income,^16^ our findings show that households affected by podo do experience catastrophic health expenditure, which is likely to cause further impoverishment, indicating the importance of prioritizing podo in the national NTD plan in order that affected households may benefit from schemes such as subsidized health insurance so they are better financially protected.

Median annual income of podo case households was significantly lower than that for control households. This difference is explained by the fact that the disability associated with podo renders those affected less economically productive, hindering the capability of affected persons to increase their income to match that of their unaffected controls. This result agrees with Tekola et al.^7^ who stated that the disability associated with podo has devastating negative effects on households’ economic productivity. On the other hand, leprosy case households’ annual income was not significantly different from that of control households. This finding is not consistent with earlier data on the effects of erythema nodosum leprosum reactions on affected households. This may be explained by the fact that leprosy has been well addressed by the CBC, which provides patients with monthly salaries for skilled and unskilled labor within the facility and also provides monthly allowances and food for the most disabled patients, thereby increasing their consumption of other goods and services.

The direct costs of commonly occurring household diseases in podo case and control households were similar at 147 USD and 142 USD, respectively. However, podo-affected households spend an extra 142 USD out of pocket yearly on podo; thus, podo-affected households spend an average of 288 USD annually, considerably higher than that spent by control households. These findings are comparable with the studies conducted in Ethiopia^7^ as follows: the direct costs in our study were 142 USD, whereas they were 143 USD in Ethiopia in 2006. They were different with the costs of Buruli ulcer treatment at Akonolinga in Cameroon,^14^ where direct out-of-pocket costs of treatment constituted 71 USD, which was catastrophic at the household level and consistent with our findings.

Annually, 36 USD and 76 USD, respectively, was spent on direct costs of common household diseases by leprosy-affected and control households. Leprosy case households did not incur extra health expenditure for leprosy treatment because there is free treatment, rehabilitation, and food for leprosy patients and families within the treatment center. This is in contrast to the large amounts spent by podo households for treatment of the disease; thus, a statistically significant difference was observed in household direct costs between podo and leprosy.

Podoconiosis-affected households experienced average indirect costs of 40 USD annually (representing 135 lost working days), whereas leprosy-affected households experienced average indirect costs of 26 USD (representing 115 working days). The overall costs (direct and indirect) to podo- and leprosy-affected households were 203 USD and 26 USD per annum, respectively. Podoconiosis-affected households suffered both out-of-pocket payments for treatment of the disease and indirect disability costs because of loss of economic productivity, whereas leprosy-affected households suffered only the disability costs associated with leprosy. One explanation for this difference is that leprosy has been incorporated into the national NTD control program and is also taken care of by other non-governmental organizations and missionary movements such as the CBC (the owners of Mbingo Baptist Hospital).

At 40%, the proportion of annual household income consumed for treatment of podo is considerably higher than the World Bank threshold for catastrophic health expenditure. It is higher than the cost burden of Buruli ulcer treatment in Cameroon (25% of household income),^14^ and comparable to that observed among leprosy-affected households in rural India (40% of household income).^8^

This study has several strengths; first, the study used comparison groups including households affected by podo or leprosy and unaffected neighboring households. This strengthens the comparison of costs incurred in different categories. Second, we have assessed both direct and indirect cost of both diseases, which enables a broader picture of their economic effects to be developed. There were, however, some limitations: household diseases were limited to those commonly occurring annually in a household. This was carried out to reduce overestimation of average household cost of diseases within the community. Indirect costs were calculated based on household hourly income and not per patient hourly income and so may be overestimated. Productivity costs were limited to economic activities, and domestic productivity was not included in the cost estimation. Number of school days lost because of disease was not captured in this study. Family income for farmers was estimated based on quantity of commodities produced and sold, but very few of the farmers can give a robust estimate of the exact quantities of commodities produced. It is worth noting that, the scope of this study was limited to leprosy patients’ resident at the leprosarium. Therefore, the direct costs of leprosy treatment observed in this study truly reflects situations under a control scheme but, might not be a true representation in the population where patients not benefiting from control schemes are anticipated to spent money for their treatment.

## CONCLUSION

Given the catastrophic out-of-pocket expenditure on treatment of podo in affected families, and the prevalence of the condition,^4^ health policy in Cameroon must prioritize prevention and treatment interventions for these households and communities. This study suggests that the economic effects of leprosy have been partly mitigated by government and nongovernment provision of treatment and rehabilitation services to patients and their families who no longer suffer catastrophic health expenditure to access treatment. Similar provision is urgently needed for podo patients. Models of disease management for people with podo have been tested in other low-resource settings, whereas disability inclusion models can be adapted from those used so successfully for people with leprosy. We call on the government of Cameroon to prioritize podo—to prevent new disease and disability and to ensure financial risk protection for affected households.
